# Nurse-performed ultrasound: a new weapon against COVID-19

**DOI:** 10.1186/s13054-020-03160-6

**Published:** 2020-07-14

**Authors:** Jianhua Sun, Qi Li, Xinjuan Wu, Xiaoting Wang, Dawei Liu

**Affiliations:** 1 Department of Critical Care Medicine, Peking Union Medical College Hospital, Chinese Academy of Medical Sciences, Beijing, 100730 China; 2 Nursing Department, Peking Union Medical College Hospital, Chinese Academy of Medical Sciences, Beijing, 100730 China

Dear Editor,

During the COVID-19 outbreak, nurses were critical to saving patients’ lives and improving medical outcomes. Because the disease was highly contagious, protective clothing and gloves were worn in nursing practice. Many procedures, such as peripheral vein puncture and blood collection, were difficult to do. Ultrasound was being performed by doctors to guide hemodynamic monitoring and lung evaluation in ICU [1]. Besides, nurses could use ultrasound to solve difficulties in nursing, such as ultrasound-guided vascular puncture, chest physical therapy, and gastric residual volume (GRV) measurement (Fig. 1). Ultrasound was making nursing easier in critically care. In this letter, we summarize the application of ultrasound in patients of COVID-19.

**Fig. 1:**
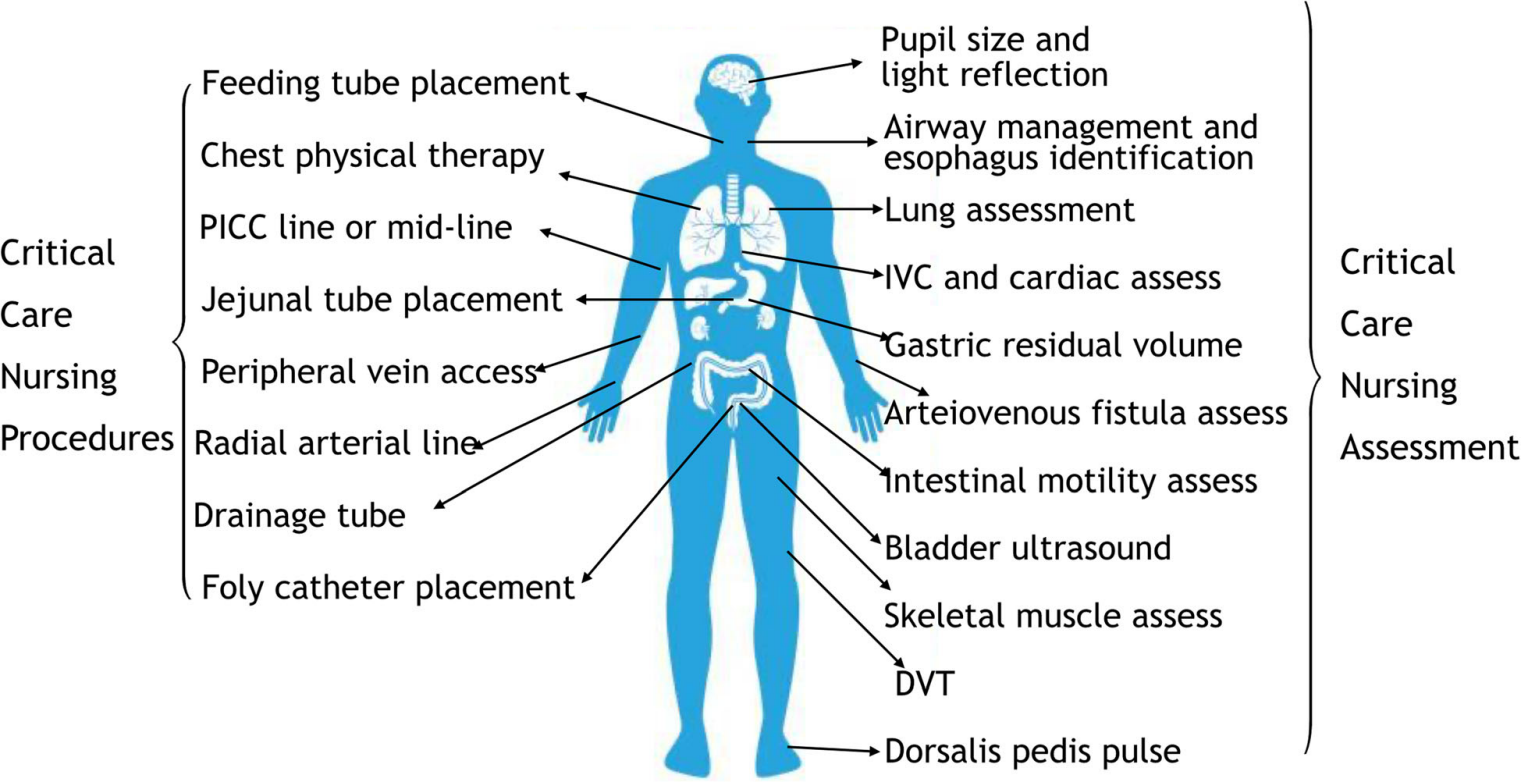
Ultrasound-guided nursing procedures and assessment in critical care

## Ultrasound: a useful tool for nursing procedures

Nurse applied ultrasound to visualize the tip of the catheter and target the vessels in real time and increase the success rate [2]. During the placement of a feeding tube, nurses can identify the esophagus and airway through ultrasound for the first time. Similarly, ultrasound was useful to the placement of the jejuna nutrition tube [3]. The application of ultrasound helps to avoid risks and optimize care procedures.

## Ultrasound: qualitative and quantitative assessments for nursing

Through ultrasound, nurses can carry out hemodynamic assessment, lung assessment, GRV measurement, thrombosis screening, and so on. Take GRV as an example. Studies reported a good correlation between the gastric antral cross-sectional area and GRV [4]. Ultrasound could dynamically assess GRV and the contraction of the gastric antrum. Ultrasound was performed to assess the patient’s condition and implement effective nursing measures.

## Ultrasound: bedside decision-making for nurses

Dyspnea was common in patients with COVID-19. Nurses play an important role in lung care. 90.5% of acute respiratory failure can be accurately diagnosed within 3 min by BLUE protocol. For patients with lung consolidation, goal-oriented chest physiotherapy could be applied based on ultrasound signs [5]. For patients with pulmonary interstitial syndrome, nurses need to pay more attention to indicators such as edema, inflow and outflow, and CVP.

In this COVID-19 outbreak, the application of ultrasound in patients involves ultrasound-led nursing assessment and ultrasound-guided nursing procedures. Ultrasound was helpful to solve existing problems in nursing practice and guide bedside decision-making. Ultrasound has now become easily available and nurses could benefit from integrating this technology into their clinical practice when taking care of the critically ill.

## Data Availability

The datasets used and analyzed during the current study are available from the corresponding author on reasonable request.
